# Bronchial microbiome of severe COPD patients colonised by *Pseudomonas aeruginosa*

**DOI:** 10.1007/s10096-013-2044-0

**Published:** 2014-01-22

**Authors:** L. Millares, R. Ferrari, M. Gallego, M. Garcia-Nuñez, V. Pérez-Brocal, M. Espasa, X. Pomares, C. Monton, A. Moya, E. Monsó

**Affiliations:** 1 Fundació Parc Taulí, Sabadell, Spain; 2 CIBER de Enfermedades Respiratorias, CIBERES, Bunyola, Spain; 3 Universitat Autònoma de Barcelona, Esfera UAB, Barcelona, Spain; 4 Fundació Institut d’Investigació Germans Trias i Pujol, Badalona, Spain; 5 Genomics and Health Area, Centro Superior de Investigación en Salud Pública—Fundación para el Fomento de la Investigación Sanitaria y Biomédica de la Comunidad Valenciana (CSISP-FISABIO), Valencia, Spain; 6 CIBER Epidemiología y Salud Pública (CIBERESP), Barcelona, Spain; 7 Department of Genetics, Institut Cavanilles de Biodiversitat i Biologia Evolutiva, (ICBiBE) Universitat de València, Valencia, Spain; 8 Department of Respiratory Medicine, Hospital Universitari Parc Taulí, Sabadell, Spain; 9 Department of Microbiology, Hospital Universitari Parc Taulí, Sabadell, Spain

## Abstract

The bronchial microbiome in severe COPD during stability and exacerbation in patients chronically colonised by *Pseudomonas aeruginosa* (PA), has not been defined. Our objective was to determine the characteristics of the bronchial microbiome of severe COPD patients colonised and not colonised by *P. aeruginosa* and its changes during exacerbation. COPD patients with severe disease and frequent exacerbations were categorised according to chronic colonisation by *P. aeruginosa*. Sputum samples were obtained in stability and exacerbation, cultured, and analysed by 16S rRNA gene amplification and pyrosequencing. Sixteen patients were included, 5 of them showing chronic colonisation by *P. aeruginosa. Pseudomonas* genus had significantly higher relative abundance in stable colonised patients (*p* = 0.019), but no significant differences in biodiversity parameters were found between the two groups (Shannon, 3 (2–4) vs 3 (2–3), *p* = 0.699; Chao1, 124 (77–159) vs 140 (115–163), *p* = 0.364). In PA-colonised patients bronchial microbiome changed to a microbiome similar to non-PA-colonised patients during exacerbations. An increase in the relative abundance over 20 % during exacerbation was found for *Streptococcus*, *Pseudomonas*, *Moraxella*, *Haemophilus*, *Neisseria*, *Achromobacter* and *Corynebacterium* genera, which include recognised potentially pathogenic microorganisms, in 13 patients colonised and not colonised by *P. aeruginosa* with paired samples. These increases were not identified by culture in 5 out of 13 participants (38.5 %). Stable COPD patients with severe disease and PA-colonised showed a similar biodiversity to non-PA-colonised patients, with a higher relative abundance of *Pseudomonas* genus in bronchial secretions. Exacerbation in severe COPD patients showed the same microbial pattern, independently of previous colonisation by *P. aeruginosa*.

**Electronic supplementary material:**

The online version of this article (doi:10.1007/s10096-013-2044-0) contains supplementary material, which is available to authorized users.

## Introduction

Sputum has traditionally been used for the identification of bronchial infection by potentially pathogenic microorganisms (PPMs) in exacerbated chronic obstructive pulmonary disease (COPD) patients, and positive cultures are found in a significant proportion of patients in this clinical setting, *Haemophilus influenzae*, *Streptococcus pneumoniae* and *Moraxella catarrhalis* being the bacteria most often recovered [1, 2]. *P. aeruginosa* is a cause of exacerbation in severe COPD patients [3–8], and studies that have collected sequential samples of bronchial secretions from patients with advanced disease have demonstrated that the acquisition of this PPM is in most cases associated with the appearance of exacerbation symptoms [9]. Advanced respiratory disease is considered a significant risk factor for *P. aeruginosa* infection when exacerbation symptoms appear, together with previous hospitalisations and antibiotics or steroid courses, and the use of antibiotic therapy targeting this PPM is recommended in current guidelines when these determinants are identified [10].

Persistence of positive cultures of bronchial secretions for *P. aeruginosa* after an exacerbation occurs in a minority of severe COPD patients, and in most cases is a transient phenomenon [11]. However, long-term persistence of *P. aeruginosa* as a chronic colonising strain has been reported in up to one fifth of COPD patients with a sputum culture positive for this PPM [9, 11, 12], and more commonly in patients who had associated bronchiectasis [13]. COPD patients with severe disease who have had previous positive cultures for *P. aeruginosa* have a worse prognosis when exacerbation symptoms appear [8], and the use of antibiotic therapy active against *P. aeruginosa* in these patients is common in current practice [12], but open to debate [14].

Currently available culture-based techniques are not suitable for the identification of up to 80 % of microorganisms inhabiting mucosal surfaces [15, 16]. Bacteria that are part of the respiratory microbiome are difficult to culture by conventional methods, and false-negative results are often found in the face of obligate anaerobes and when bacterial loads are low [17]. The application of pyrosequencing to PCR-amplified 16S rRNA gene has taken the study of microbial diversity to an unprecedented level of detail [18–20]. Culture-independent microbiological techniques have demonstrated that the bronchial tree is not sterile during health and have documented significant changes in the respiratory microbiome in chronic lung diseases [21]. In this study, we aimed to determine the differences in the bronchial microbiota in severe COPD patients with chronic colonisation by *P. aeruginosa* and in patients non-colonised by this PPM and the change of this microbiota when an exacerbation appears, identifying the microorganisms that overgrow during the episode. Bronchial secretions were examined by amplification and pyrosequencing of the 16S rRNA gene, and patients with and without chronic colonisation by this PPM were compared, both in stability periods and during exacerbation.

## Materials and methods

### Design and population

We enrolled a cohort of stable severe COPD outpatients who had been diagnosed according to the criteria of the Global Initiative for Chronic Obstructive Lung Disease (GOLD) [22] and who had reported more than two exacerbations per year, between January and December 2010. Patients were examined at baseline in a stable condition from >8 weeks, and their sociodemographic data, smoking habits, respiratory symptoms, treatments, sputum microbiological characteristics and lung function were recorded. Participants were followed for 6 months after this baseline assessment, and follow-up visits were scheduled every 3 months, or when exacerbation symptoms appeared. Every visit included the recovery of spontaneous sputum samples that were processed for microbiological cultures and culture-independent microbiological techniques. Detailed exclusion criteria are provided in the online supplementary file. Acute episodes of increased breathlessness, sputum production and/or purulence appearing during follow-up and treatment with antibiotics and/or oral corticosteroids by a physician were considered exacerbations [23]. Patients with positive cultures for *P. aeruginosa* in three or more consecutive sputum cultures obtained during a period of 6 months before enrolment in the study were considered to be chronically colonised by this PPM [24, 25]. The present study was reviewed and approved by the local research ethics committee in Catalonia, Spain, and written informed consent was obtained from all subjects.

### Clinical variables, sputum collection and microbiology

Sociodemographic data were recorded at recruitment. Patients answered an epidemiological questionnaire that covered smoking habits, respiratory symptoms, previous exacerbations and treatments. Spontaneous sputum was collected from every patient under clinical stability and during exacerbation, before the administration of antibiotic therapy. Details on the clinical variables and microbiological processing are provided in the supplementary file.

### Samples and DNA extraction

Sputum samples diluted with dithiothreitol were incubated at 37 °C for 15 min and centrifuged for 10 min at 4 °C. The pellet was resuspended in 1 ml of a lysis buffer containing 100 U/ml mutanolysin, 47,700 U/ml lysozime and 2 U/ml lysostaphin, and was incubated for 30 min at 37 °C. DNA was extracted using a QIAamp DNA Blood Midi kit (Qiagen, Helden, Germany) according to the manufacturer’s instructions and eluted in 200 μl of sterile water. DNA was quantified in the Nanodrop ND-1000 Spectrophotometer (NanoDrop Techologies, Wilmington, DE, USA) and stored at –80 °C.

### PCR amplification of the V1–V3 region of the 16S rRNA gene

The hypervariable regions V1, V2 and V3 of the 16S rRNA gene were amplified with E8 forward (AGAGTTTGATCMTGGCTCAG) and B530 reverse (CCGCGGCKGCTGGCAC) primers and with 12 different barcodes. The PCRs were carried out in a volume of 40 μl with Biomix (Bioline, London, UK) and 10 mM of each primer, and were performed on the Mastercycler® pro thermal cycler (Eppendorf, Hamburg, Germany). After amplification, the products were visualised in 2 % agarose gels. Amplified product was purified using NucleoFast® 96 PCR Clean-Up kit (Macherey-Nagel, Düren, Germany), eluted in 28 μl of PCR-grade water and quantified with Quant-iT PicoGreen dsDNA Assay Kit (Invitrogen, Life Technologies, Carlsbad, CA, USA). Twelve samples with different barcode sequences were pooled in equimolar amounts into a single tube and pyrosequencing was carried out using Roche 454 GS-FLX System Titanium Chemistry.

### Library analysis sequences and microbiome accession numbers

16S rRNA raw sequences were analysed using the mothur software package 1.27 [26] to remove sequences that were less than 200 bp or greater than 520 bp in length and chimeras. The Quantitative Insights into Microbial Ecology (QIIME) pipeline [27] was used for sequence processing to obtain taxonomic information. A taxonomic classification was performed and operational taxonomic units (OTUs) present in the samples at 97 % identity were determined.

Bacterial 16S rDNA data sets from this study are accessible in the European Nucleotide Archive under the study accession number PRJEB4144, available at http://www.ebi.ac.uk/ena/data/view/PRJEB4144, with the sample accession numbers ERS255709–739.

### Statistical analysis

Statistical analyses were performed using SPSS statistical software package version 18 (SPSS, Chicago, IL, USA), R package (http://www.r-project.org), using the VEGAN library to cluster and construct abundance heat maps and LEfSe to identify differences between groups [28]. Results for categorical variables are expressed as absolute and relative frequencies and results for continuous variables as means and standard deviations (SD), or as medians and percentiles 25–75 (P25–P75) when the distribution was not normal.

Bacterial biodiversity was assessed through the Chao1 estimator [29] and Shannon [30] index, which estimate the richness and homogeneity of the microbiome respectively. Both indices were calculated after subsampling with QIIME to avoid sequencing effort bias. The beta-diversity Bray–Curtis dissimilarity index [31] was used to make principal correspondence analysis.

Variability of the bacterial flora between consecutive samples of the same patient was expressed as a percentage of change of relative abundance (relative abundance consecutive sample – relative abundance baseline sample × 100). Baseline variability for all genera was calculated by the comparison of consecutive samples obtained during stability from the same patient, to establish the expected variability range under stable conditions for severe COPD patients. Variability of the bacterial flora when exacerbation appears was equally expressed as percentage of change of relative abundance, using the previous baseline sample as the reference (relative abundance exacerbation sample – relative abundance baseline sample × 100). Statistical tests used in the study were two-sided, and a *p* value of 0.05 or less was reported to be statistically significant.

## Results

### Patients’ characteristics

Sixteen severe COPD patients, 6 of them at GOLD stage III (37.5 %) and 10 at stage IV (62.5 %), were included in the study. All of them were former smokers with an average age of 71 (SD 6) years (Table 1). Five patients had criteria of chronic *P. aeruginosa* colonisation, and participants were classified into two groups according to colonisation by this PPM. Microbiological data of both groups were compared and the variability in exacerbation was assessed for PA-colonised and non-PA-colonised patients, using the previous available results in stability as the reference.

**Table 1:** Patients’ characteristics

Patients	Total	PA-colonised	Non-PA-colonised
*n*	16	5	11
Age (years), mean (SD)	71 (6)	72 (7)	70.5 (6)
Male, *n* (%)	16 (100)	5 (100)	11 (100)
Smoking (pack-years), median (IQR)	57 (57–110)	100 (50–110)	50 (40–80)
FEV_1_ post-BD (% predicted), mean (SD)	36 (30–40)	41 (30–48)	34 (30–37)
Dyspnoea scale, mean (SD)	2.25 (0.6)	2.2 (0.4)	2.3 (0.6)
BMI (kg/m^2^), mean (SD)	28 (4)	28 (4)	28 (4)
6MWD (m), median (IQR)	240 (140–305)	300 (220–310)	230 (115–292)
BODE index, median (IQR)	5 (4.5–7.5)	5 (4–5)	6 (5–8)
Inhaled β2-adrenergics, *n* (%)	16 (100)	5 (100)	11 (100)
Inhaled anticholinergics, *n* (%)	16 (100)	5 (100)	11 (100)
Inhaled corticosteroids, *n* (%)	16 (100)	5 (100)	11 (100)
Positive PPM culture in stability, *n* (%)	12 (86)	5 (100)	7 (64)
Chronic colonisation by *P. aeruginosa*, *n* (%)	5 (31)	5 (100)	0

No statistically significant differences between PA-colonised and non-PA-colonised patients for any variable

*SD* standard deviation; *IQR* interquartile range; *FEV*_*1*_ *post-BD* post-bronchodilator forced expiratory volume in 1 s; *BMI* body mass index; *6MWD* 6-min walking distance; *BODE index* body mass index, airflow obstruction, dyspnoea, and exercise capacity index; *PPM* potentially pathogenic microorganism

### Bronchial microbiome

Microbiological cultures were performed in sputum samples obtained during stability (*n* = 14) and exacerbation periods (*n* = 15). Cultures were positive for PPMs in 12 stability (86 %) and 10 exacerbation samples (67 %), *P. aeruginosa* and *H. influenzae* being the most frequent isolated PPMs in the studied patients (Table 2). Culture-independent analyses were also performed in all sputum samples obtained during stability and exacerbation and the average number of sequences per sample obtained after filtering was 4,695. Most prevalent phyla in bronchial microbiome were *Proteobacteria* (50 %) and *Firmicutes* (31 %), followed by *Actinobacteria* (8 %), *Bacteroidetes* (6 %) and *Fusobacteria* (3 %). One hundred twenty-eight different OTUs were obtained at genus level (supplementary file: Figure 1), and after removing the OTUs present in only one sample, the remaining ones were used for further analysis.

**Table 2:** Sputum cultures under stability and exacerbation

	Stable	Exacerbated
*n*	14	15
Positive culture for PPM, *n* (%)	12 (86)	10 (67)
Polymicrobial cultures, *n* (%)	4 (29)	3 (20)
Microorganisms		
*Pseudomonas aeruginosa*	5 (42)	3 (30)
*Haemophilus influenza*	4 (33)	4 (40)
*Moraxella catarrhalis*	2 (17)	1 (10)
*Streptococcus pneumonia*	2 (17)	1 (10)
*Escherichia coli*	1 (8)	–
*Alcaligenes* spp.	1 (8)	1 (10)
*Staphylococcus aureus*	–	1 (10)
*Haemophilus parainfluenzae*	–	1 (10)
*Stenotrophomonas maltophilia*	–	1 (10)

**Fig. 1:**
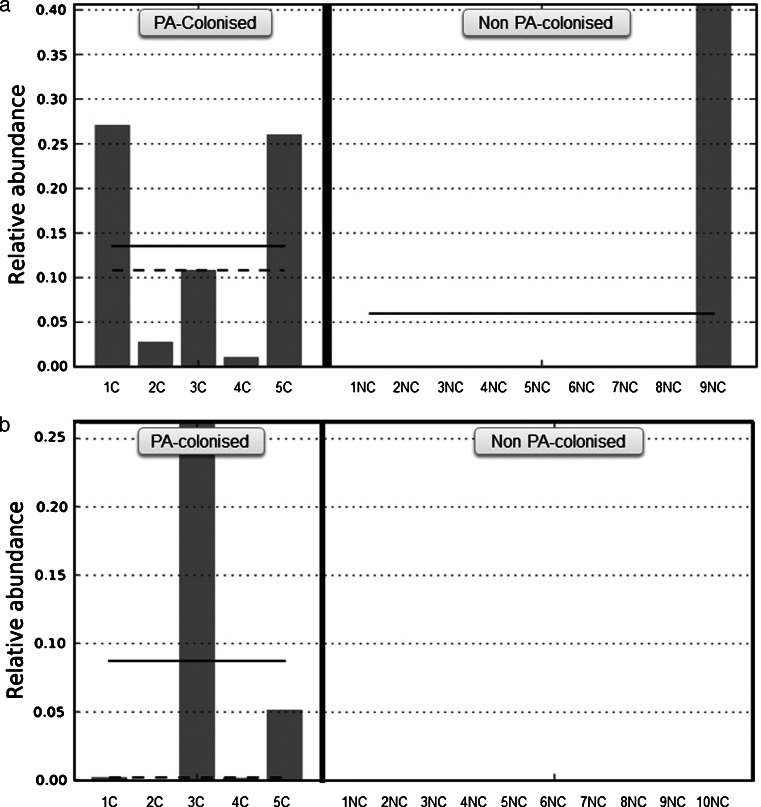
Relative abundance of *Pseudomonas* genus ** a** under stability and** b** during exacerbation in PA-colonised and non-PA-colonised patients. *Solid line* represents the mean and *dashed line* the median

The relative abundance of specific genera in samples from PA-colonised and non-PA-colonised patients were compared, during both stability and exacerbation periods, to assess the effect of this colonisation on the remaining bronchial flora. Statistically significant differences were only found in the *Pseudomonas* genus, which had a higher relative abundance in PA-colonised patients in both the stable and the exacerbated state (Fig. 1, *p* = 0.019 and *p* = 0.003 respectively, Mann–Whitney *U* test). The biodiversity of bacterial communities in sputum samples obtained during stability measured with Chao1 richness estimator and Shannon index value showed no statistically significant differences between PA-colonised and non-PA-colonised groups (Shannon index, 3 (2–4) vs 3 (2–3), *p* = 0.669; Chao index, 124 (77–159) vs 140 (115–163), *p* = 0.364; Mann–Whitney *U* test). Similar results were obtained for the samples obtained during an exacerbation (Shannon index, 4 (2–4) vs 4 (2.5–5), *p* = 0.595; Chao index, 165 (93–196) vs 163 (70–193), *p* = 0.679, Mann–Whitney *U* test).

**Fig. 2:**
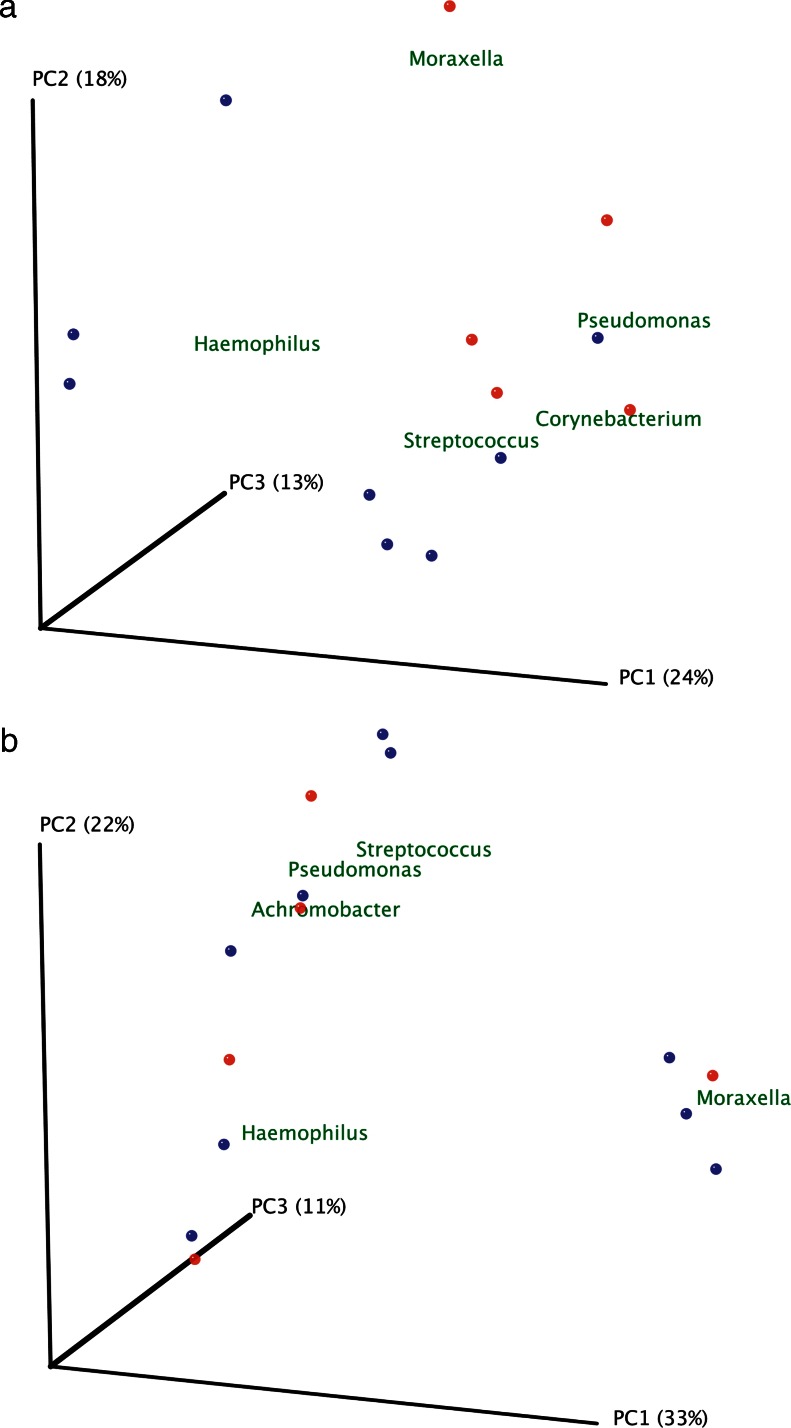
Principal coordinate analysis with Bray–Curtis dissimilarity index. **a** Samples from stability and **b** samples of exacerbation. *Red dots* represent colonised patients, and *blue dots* non-PA-colonised patients

**Fig. 3:**
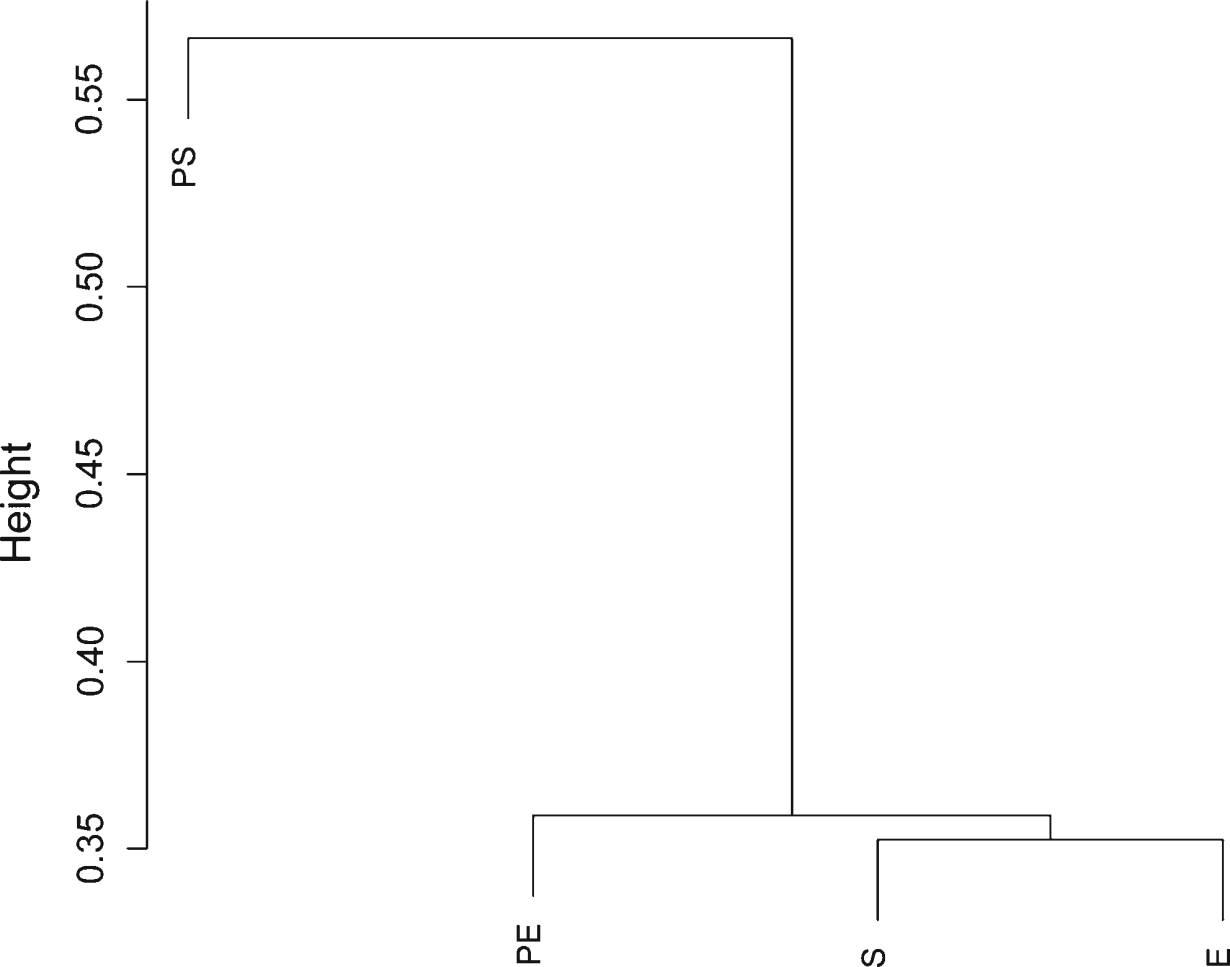
Cluster dendrogram with Bray–Curtis dissimilarity index. Samples were combined depending on their clinical situation and clustered with the Bray–Curtis index, which takes values between 0 and 1 (0 meaning that samples share all the genera and 1 meaning samples do not share any). *PS* colonised by *P. aeruginosa* under stability; *PE* colonised by *P. aeruginosa* during exacerbation; *S* non-colonised by *P. aeruginosa* under stability; *E* non-colonised by *P. aeruginosa* during exacerbation)

**Fig. 4:**
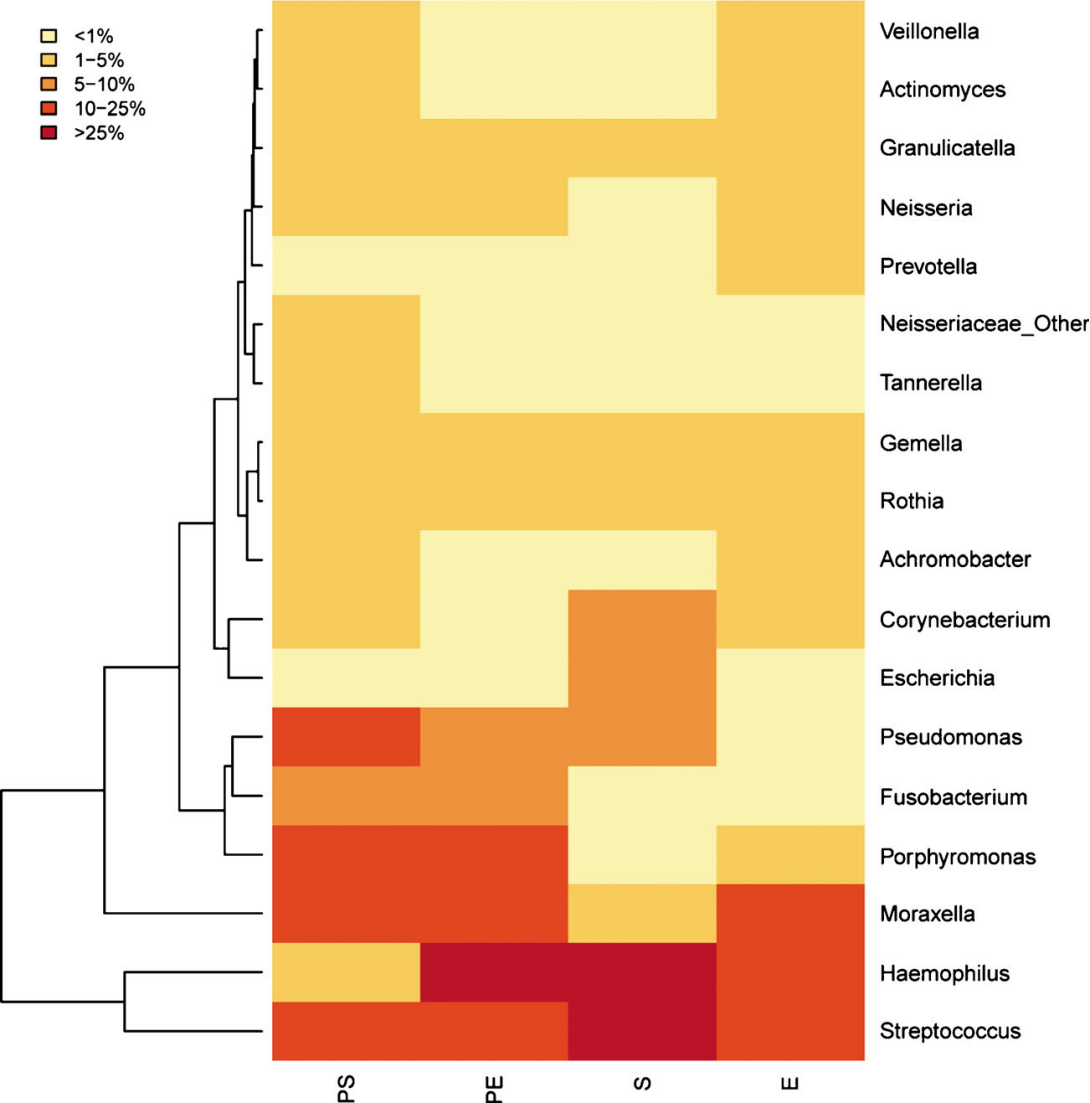
Heat map showing the most abundant genera in the four groups of samples. Columns represent the groups and rows the genera whose relative abundance is >1 % in at least one sample. The relative abundance of each genus is represented by the colour key

Rarefaction curves were calculated for PA-colonised and non-PA-colonised patients using the observed species metric (supplementary file: Figure 2) and showed that the number of sequenced taxa was enough to estimate total population diversity in both groups. Differences between microbial communities in both groups of patients were assessed by principal coordinates analyses with the Bray–Curtis beta-diversity metric, with samples obtained during stable (Fig. 2a) and exacerbation periods (Fig. 2b). Genera with the highest relative abundance were plotted in this analysis, and two clusters were observed in samples obtained during a stability period, corresponding to PA-colonised or non-PA-colonised samples, with *Pseudomonas*, *Corynebacterium* and *Moraxella* as the most abundant genera in colonised patients. During exacerbation, samples from PA-colonised and from non-PA-colonised patients did not cluster separately. The Bray–Curtis metric was also used after pooling all patients for clustering together patients colonised by *P. aeruginosa* during stability (PS) and exacerbation (PE); and stable (S) and exacerbated (E) non-PA-colonised COPD patients, according to genera composition (Fig. 3). In this analysis the bacterial community obtained during an exacerbation in PA-colonised patients showed a closer similarity to the microbiome of non-PA-colonised severe COPD participants, in both stable and exacerbated states, than to the microbiome recovered from PA-colonised patients during their stability periods. When most abundant genera in the four clinical situations were represented in a heat map, a range of genera showed low relative abundance but high prevalence, which included *Veillonella*, *Actinomyces*, *Granulicatella*, *Neisseria*, *Prevotella*, *Tannerella*, *Gemella*, *Rothia* and *Achromobacter*, with *Streptococcus* as the most abundant genus in all clinical situations (Fig. 4). *Haemophilus*, *Moraxella* and *Pseudomonas* genera, which include the main PPM in COPD, were also frequently recovered, but showed a high variability among the four assessed clinical situations. *Pseudomonas* genus was the most common in stable PA-colonised patients, *Haemophilus* being the genus more often found in non-PA-colonised patients and during exacerbation, independently of the previous history of *P. aeruginosa* colonisation.

### Variability in bronchial microbiome during exacerbation

**Fig. 5:**
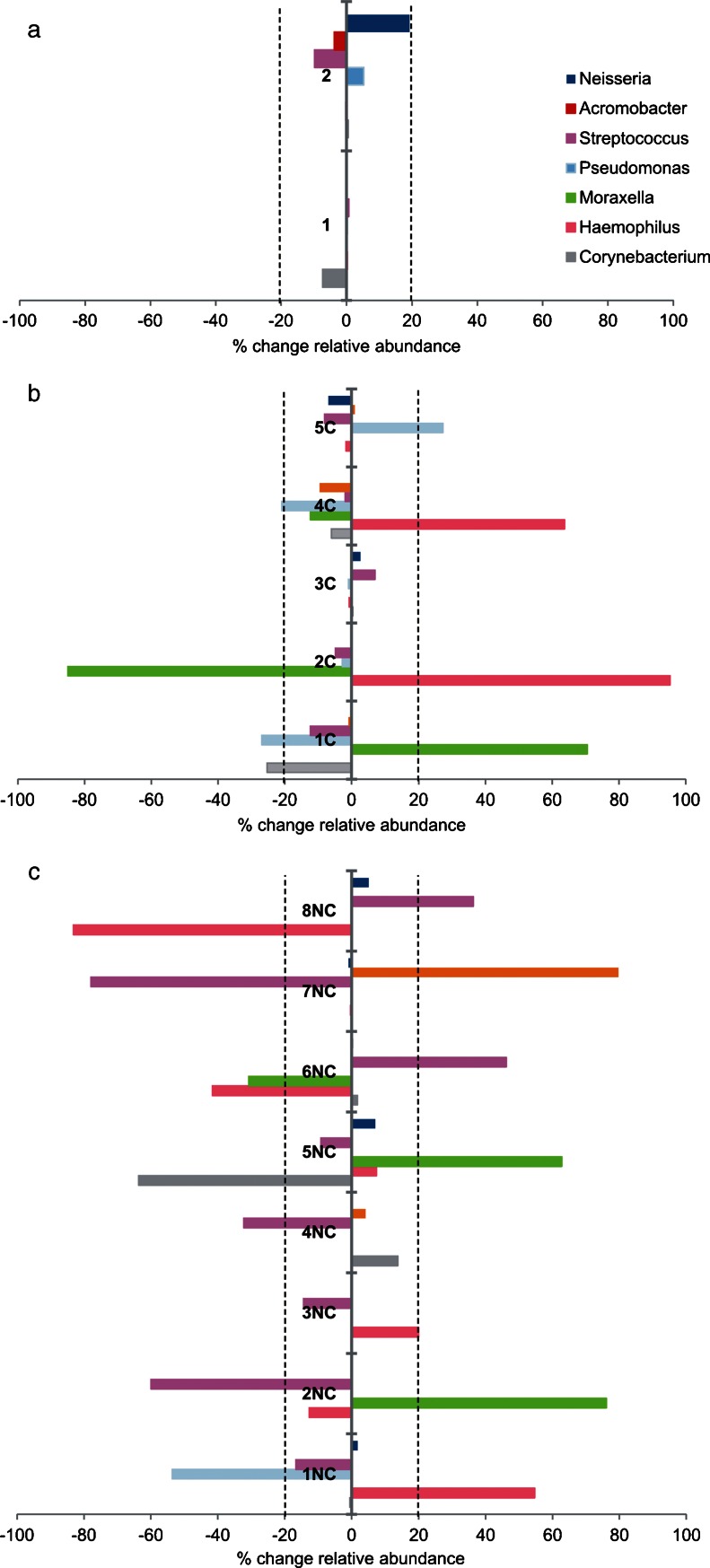
Percentage of change in the relative abundance of genera. Only genera with a percentage of change over 20 % in at least one patient are represented. **a** Percentage of variability in the genera observed in consecutive samples recovered in stable situation (*n* = 2); **b** Percentage of variability in exacerbation in patients colonised by *P. aeruginosa* using the baseline stability sample as the reference (*n* = 5); **c** Percentage of variability in exacerbation in non-PA-colonised patients (*n* = 7). *Dark blue*: *Neisseria*; *orange*: *Achromobacter*; *purple*: *Streptococcus*; *light blue*: *Pseudomonas*; *green*: *Moraxella*; *pink*: *Haemophilus*; *grey*: *Corynebacterium*

Baseline variability of the bronchial microbiome in severe COPD was determined calculating the percentage of variation for all examined genera in two sputum samples obtained consecutively (>2 weeks) during stability from 2 patients (relative abundance consecutive sample – relative abundance baseline sample × 100), and was below 20 % for all the genera studied (*n* = 96; Fig. 5a). Changes over this value were accordingly considered abnormal for subsequent analysis. The same approach was followed for the calculation of the variability of every genus in the bronchial flora when exacerbation appears in 13 patients with paired samples from stability and exacerbation. Genera with increases over 20 % in exacerbations included *Streptococcus*, *Pseudomonas*, *Moraxella*, *Haemophilus*, *Neisseria*, *Achromobacter* and *Corynebacterium*, all of which include recognised PPMs. The increase observed for specific genera when exacerbation symptoms appear paralleled a decrease of the same magnitude in the relative abundance for genera unrelated to this exacerbation, without major changes in the remaining bronchial microbiota. Exacerbation-related variability in PA-colonised and non-PA-colonised patients was similar, and did not show statistically significant differences in the genera that showed increases in their relative abundance over 20 % (*p* >0.05, Chi-squared test; Fig. 5b, c).

Increases over 20 % in specific genera identified by 16SrRNA gene pyrosequencing were compared with culture results during exacerbation in the same sample, in order to determine the sensitivity of microbiological cultures for detecting the identified changes in the bronchial microbiome. In 5 out of 13 patients (38.5 %) microbial cultures did not show PPMs whose genera increased over 20 % from baseline at pyrosequencing. Three of these patients had negative sputum cultures during exacerbation and 1 patient showed a positive culture for *P. aeruginosa,* while an increase over 20 % was detected for *Moraxella* genus at pyrosequencing without significant changes in *Pseudomonas* genus abundance. In the fifth patient, an increase in *Achromobacter* genus was shown by the culture-independent technique, while the sputum culture was positive for *Stenothropomonas maltophilia* and *Alcaligenes* spp. In 2 exacerbated patients positive sputum cultures showed a PPM previously recovered during stability. These patients were considered to be colonised, and their pyrosequencing results did not show an increase in abundance over 20 % for any genera (Table 3).

**Table 3:** Changes in the microbiome during exacerbation detected by traditional cultures and 16S pyrosequencing. Only the increases above 20 % are detailed in the pyrosequencing data

Patients	Standard culture positive	16S pyrosequencing abundance increase >20 %	16S pyrosequencing additional relevant information
Colonised patients			
1	*P. aeruginosa*	↑ *Moraxella*	PPM not identified by culture
2	*H. influenzae*	↑*Haemophilus*	No additional information
3	*P. aeruginosa*	Only changed <20 %	Colonising *P. aeruginosa*
4	*H. influenzae*	↑*Haemophilus*	No additional information
5	Negative	↑*Pseudomonas*	PPM not identified by culture
Non-PA-colonised patients			
1	*H. parainfluenzae*	↑*Haemophilus*	No additional information
2	*H. influenzae*	↑*Moraxella*	No additional information
	*M. catarrhalis*		
3	*H. influenzae*	↑*Haemophilus*	No additional information
4	*S. pneumoniae*	Only changed <20 %	Colonising *S. pneumoniae*
5	Negative	↑*Moraxella*	PPM not identified by culture
6	*S. pneumoniae*	↑*Streptococcus*	No additional information
7	*S. maltophilia*	↑*Achromobacter*	PPM not identified by culture
	*Alcaligenes* spp.		
8	Negative	↑*Streptococcus*	PPM not identified by culture

## Discussion

This study analysed the microbiome of severe COPD patients through amplification and pyrosequencing of the 16S rRNA gene. Assessing sputum samples obtained during stability from patients colonised by *P. aeruginosa* and non-PA-colonised patients, we observed that the microbiome of PA-colonised COPD patients showed an expected increase in the *Pseudomonas* genus. Differences in the bronchial microbiome between PA-colonised and non-PA-colonised patients disappeared during exacerbation, a clinical situation that prompted a change in the microbiome of patients chronically colonised by this PPM to a bacterial flora equivalent to that found in exacerbated non-PA-colonised patients. Besides, we found that some genera increased their relative abundance during exacerbation, including *Streptococcus*, *Pseudomonas*, *Moraxella*, *Haemophilus*, *Neisseria*, *Achromobacter* and *Corynebacterium*, all of which include recognised PPMs, and this change was equivalent in PA-colonised and non-PA-colonised patients. Simultaneous sputum cultures did not identify genera increasing their abundance in one third of the exacerbations appearing in severe COPD patients.

We found that the bronchial tree of severe COPD patients shows a wide range of prevalent genera, in agreement with previous reports in healthy subjects and patients with moderate disease [18, 19, 32]. Thus, the bacterial diversity in the lower airway in severe COPD individuals without signs of infection was much higher than previously anticipated, with a complex bacterial community that includes *Streptococcus, Haemophilus, Moraxella, Veillonella*, *Actinomyces*, *Granulicatella*, *Neisseria*, *Prevotella*, *Tannerella*, *Gemella*, *Rothia* and *Achromobacter* as the main recovered genera. *Pseudomonas* was the most common genus in stable patients who have shown previous colonisation by *P. aeruginosa*, and *Haemophilus* is the genus more often found in non-PA-colonised severe patients.

In spite of the chronic presence of *P. aeruginosa* in the bronchial tree of a subgroup of severe COPD patients, we found no statistically significant differences in Chao1 and Shannon biodiversity parameters between PA-colonised and non-PA-colonised patients, suggesting that the presence of *P. aeruginosa* in the respiratory tract of these patients does not modify the diversity of the bronchial microbiome. The conservation of a diverse microbiota is related to epithelial integrity, immunoregulation and colonisation resistance [19, 33–35] and shifts in the microbial community composition may compromise respiratory health and contribute to disease progression. Recent studies based on culture-independent sequencing technologies have reported well-defined microbiome changes in the mucosa of diverse chronic diseases, such as cystic fibrosis and inflammatory bowel diseases, among others [36]. The lack of differences in the bronchial microbiome observed in PA-colonised and non-PA-colonised patients in our study suggests that the bronchial colonisation by this PPM is not associated with the appearance of significant alterations in the bronchial flora in severe COPD patients.

We found that the bacterial community obtained during exacerbation in patients colonised by *P. aeruginosa* changes to a flora showing a close similarity with the microbiome found in non-PA-colonised severe COPD patients during exacerbation, suggesting that acute episodes in PA-colonised patients might be attributable to common PPMs instead of the colonising PPM. In severe COPD, *P. aeruginosa* may be recovered from sputum cultures obtained during stability periods [3–8]. Patients with persistent colonisation by *P. aeruginosa* have a worse prognosis than non-colonised patients when exacerbation symptoms appear [8], and require prompt treatment. Accordingly, antibiotic treatments targeting *P. aeruginosa* are currently recommended for severe patients with frequent episodes of exacerbation with this clinical profile [10]. In adults with COPD this approach is justified by the features that this disease shares with cystic fibrosis, in which *P. aeruginosa* causes chronic colonisation and is associated with significant morbidity and mortality [37–39]. Extrapolating observations from cystic fibrosis to COPD must be done with caution, however, because the carriage patterns of *P. aeruginosa* differ substantially in the two diseases [11,  40, 41]. Our findings suggest that exacerbation symptoms in PA-colonised patients are mainly related to PPMs that are a frequent cause of exacerbation in non-PA-colonised COPD patients rather than *P. aeruginosa* infection.

The variability in the abundance of genera in bronchial secretions observed during stability and exacerbation periods was assessed in our study, and stable severe COPD patients showed variabilities below 20 % for all genera in the absence of exacerbation symptoms. When exacerbation appears, this value increases well above the stability reference level for genera such as *Haemophilus, Moraxella* and *Streptococcus*, all of which include pathogenic species. These findings confirm that most cases of infectious exacerbation in severe COPD patients are due to PPMs, which are a common cause of acute symptoms in COPD, without significant differences between PA-colonised and non-PA-colonised patients. This finding suggests that antibiotic treatments prescribed for exacerbations in PA-colonised patients need to target common PPMs instead of the colonising PPM in the first instance. Interestingly, the increases in relative abundance observed in specific genera during exacerbation periods in the present study paralleled clear decreases of a similar magnitude in other genera that also included PPMs, without significant changes in the baseline flora found in the bronchial tree, again with a similar pattern in PA-colonised and non-PA-colonised patients.

The exacerbation-related changes identified in the bronchial microbiome during exacerbation were not identified by simultaneous microbiological cultures in one third of the patients in our study. Although traditional microbiological culture is the gold standard technique for the identification of bronchial colonisation [42], it has some limitations. A significant number of microorganisms do not grow in selective cultures, and fast-growing bacteria can mask other clinically important bacteria in the sample [43]. PCR amplification of 16S rRNA gene identifies the real composition of the lung microbiome [42] and can help us to understand the association between the microbiome changes and the acute stages of the disease.

Limitations of the study are the sample size, the use of sputum samples for the assessment of bronchial secretions and the absence of testing for viral infection. Enrolled patients were representative of the hospital-managed population with severe COPD, and did not suffer from severe comorbidities. Sputum was used to examine the bronchial microbiome because these samples can be obtained easily and non-invasively and the guidelines for the procedure include quality criteria. Although the sputum may be contaminated by bacterial flora of the oropharynx and the oral cavity, the microbiome composition that we have found in severe COPD patients is quite similar to that of previous reports in healthy controls and COPD patients based on the use of bronchial brushing and bronchoalveolar lavage for the sampling of bronchial secretions [18, 19]. We assumed, accordingly, that sputum is a representative sample for the study of the microbiome of bronchial secretions in severe COPD patients, with the additional advantage that the results obtained can be easily correlated with sputum culture. Viral infection in COPD patients can modify the bronchial microbiome [44] and changes in the microbiome detected during exacerbation may be partly influenced by the coexistence of infective virus in the bronchial tree.

## Conclusions

Our data confirm that a rich bronchial microbiome is found in bronchial secretions from stable COPD patients with severe disease, which includes genera with well-known PPMs that are unusual in the normal population. In severe patients colonised by *P. aeruginosa* the bacterial community showed differences with regard to non-colonised patients in their stability periods, but at times of exacerbation their microbial profile changed to a flora that included PPMs and was equivalent to the bronchial microbiome found in non-colonised patients during exacerbation. These findings argue against the need for differentiated antibiotic approaches to exacerbation in severe COPD patients colonised by *P. aeruginosa*, a suggestion that must be confirmed in larger cohorts of patients.

## Electronic supplementary material

Below is the link to the electronic supplementary material.

ESM 1 (PDF 118 kb)
