# Supplementary material for: Bronchial microbiome of severe COPD patients colonised by Pseudomonas aeruginosa
Source: Eur J Clin Microbiol Infect Dis. 2014 Jan 22;33(7):1101–11. doi: 10.1007/s10096-013-2044-0 (PMC4042013; doi:10.1007/s10096-013-2044-0)
Supplement: Supplementary file 1 — (PDF 118 kb) [file 10096_2013_2044_MOESM1_ESM.pdf]

Online supplementary file

Bronchial microbiome of severe COPD patients colonised by *Pseudomonas aeruginosa*.

Laura Millares, Rafaela Ferrari, Miguel Gallego, Marian Garcia-Nuñez, Vicente Pérez-Brocal, Mateu Espasa, Xavier Pomares, Concepción Monton, Andrés Moya, Eduard Monsó.

**Methods**

Design and population

Exclusion criteria were age <40 yrs, hospital admission for respiratory symptoms in the previous six months, diagnosis of asthma, cystic fibrosis, neoplasia or bronchiectasis, and/or treatment with oral corticosteroids or immunosuppressive drugs for any reason. Patients who changed their smoking habits and bronchodilator treatment during follow-up were also excluded.

Clinical variables

Functional characteristics were assessed at baseline and included forced spirometry and reversibility testing, six minute walking test [1], body mass index (BMI) and BODE index [2]. Forced vital capacity and $FEV_1$ were measured with the same dry rolling seal spirometer (Sibelmed, Sibelgroup, Barcelona, Spain), and expressed as absolute values (mL) and percentages of the reference values obtained from age- and height-adjusted selected volunteers from the Barcelona province [3].

Microbiology

Sputum appearance was determined according to the Murray-Washington criteria [4]and samples with >25 leucocytes per field were considered indicative of a neutrophilic inflammatory response and suitable for culture [5]. Sputum samples were then weighed, processed with an equal volume of a 1:10 dilution of dithiothreitol (DTT) (Sputasol, Oxoid Ltd., Hants, UK), and cultured according to standard methods [6]. Microbiological processing included determination of microbial typology and load through serial dilutions and culture in selective media for PPMs,

with quantitative cultures expressed as colony-forming units (cfu) per milliliter. A positive sputum culture for PPMs at a concentration of $10^2$ colony-forming units (cfu) per milliliter (mL) or greater was considered positive, and following Cabello *et al* [7], PPMs were defined as those microorganisms recognized as agents causing respiratory infections, regardless of whether they belonged to the gastrointestinal or oropharyngeal flora. Remaining sputum after standard culture was stored at -80ºC for further determinations.

## Results

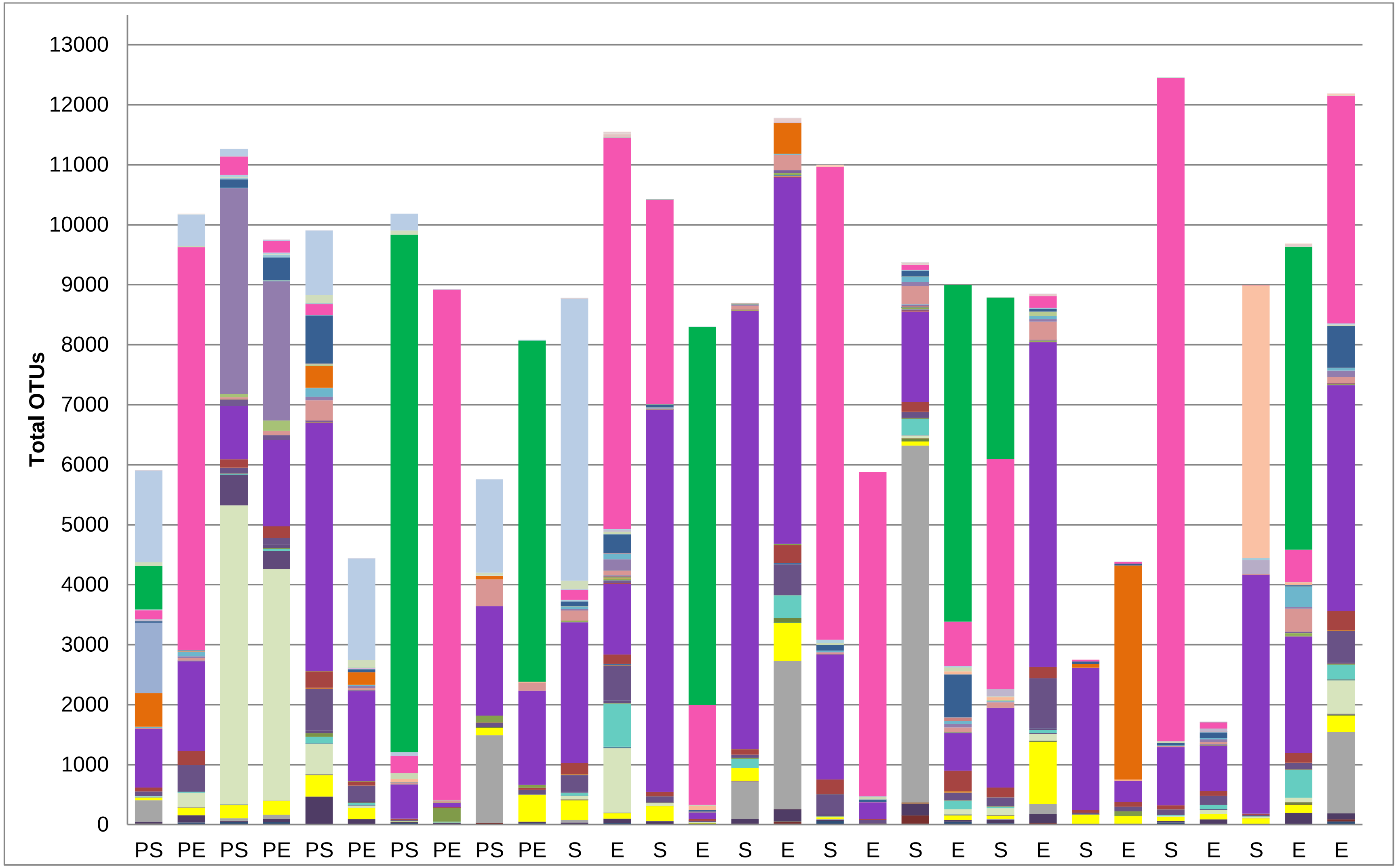
Total OTUs
13000
12000
11000
10000
9000
8000
7000
6000
5000
4000
3000
2000
1000
0
PS PE PS PE PS PE PS PE PS PE S E S E S E S E S E S E S E S E S E E

Figure 1. Abundance of the detected OTUs at genus level in the samples analysed before removing OTUs present in only one sample. (PS, colonised by *P. aeruginosa* in stability; PE colonised by *P. aeruginosa* in exacerbation; S, non-PA-colonised stability; E, non-PA-colonised in exacerbation).

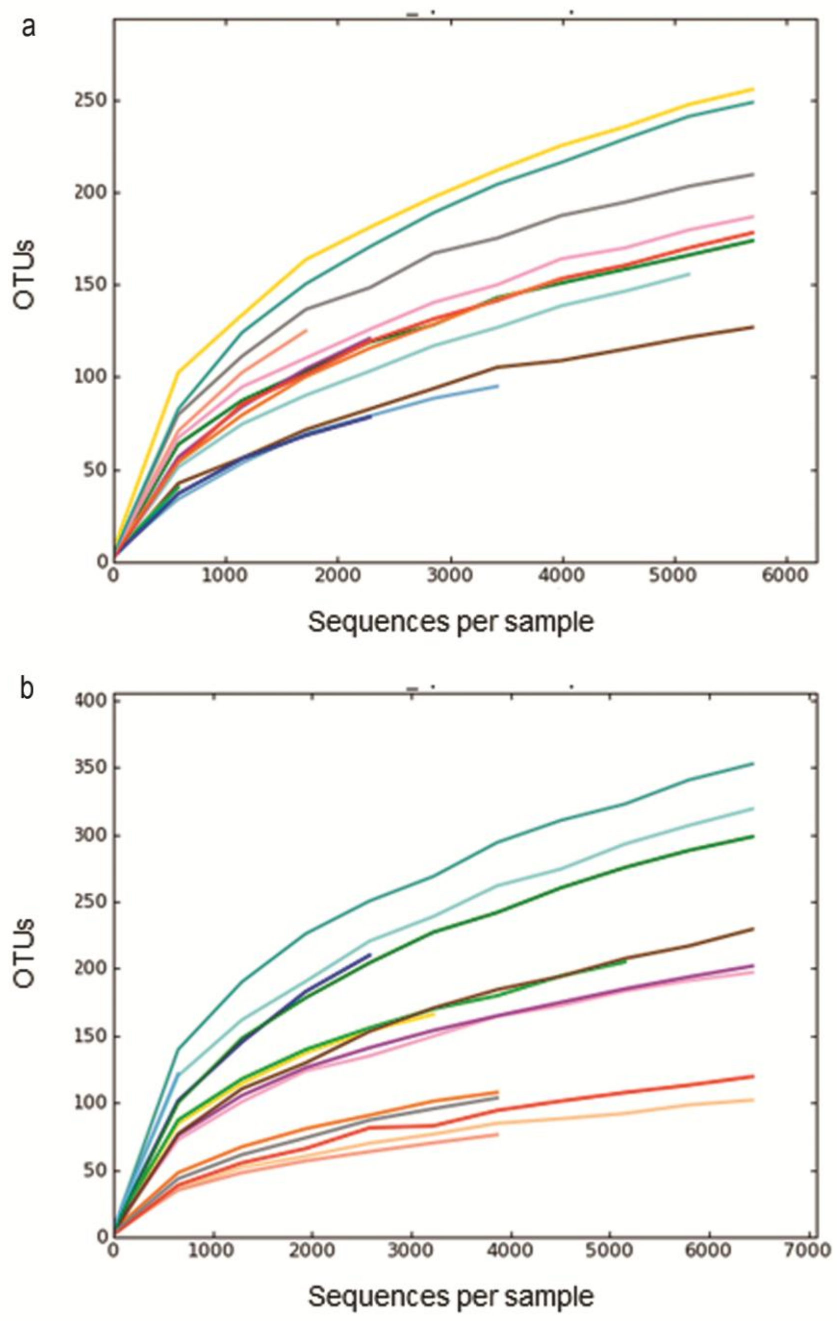


Figure 2. Rarefaction curves from stable (a) and exacerbated (b) samples. Each curve represents one sample and indicates the count of single OTUs found in this sample at each sequencing depth.

## References


1. **Standardization of Spirometry, 1994 Update. American Thoracic Society**. *Am J Respir Crit Care Med* 1995, **152**(3):1107-1136.
2. Celli BR, Cote CG, Marin JM, Casanova C, Montes de Oca M, Mendez RA, Pinto Plata V, Cabral HJ: **The body-mass index, airflow obstruction, dyspnea, and exercise capacity index in chronic obstructive pulmonary disease**. *N Engl J Med* 2004, **350**(10):1005-1012.
3. Roca J, Sanchis J, Agusti-Vidal A, Segarra F, Navajas D, Rodriguez-Roisin R, Casan P, Sans S: **Spirometric reference values from a Mediterranean population**. *Bull Eur Physiopathol Respir* 1986, **22**(3):217-224.
4. Murray PR, Washington JA: **Microscopic and baceriologic analysis of expectorated sputum**. *Mayo Clin Proc* 1975, **50**(6):339-344.
5. Van Scoy RE: **Bacterial sputum cultures. A clinician's viewpoint**. *Mayo Clin Proc* 1977, **52**(1):39-41.
6. Balows A: **Manual of Clinical Microbiology**, 5th edn: Washington DC, American Society of Microbiology 1991.
7. Cabello H, Torres A, Celis R, El-Ebiary M, Puig de la Bellacasa J, Xaubet A, Gonzalez J, Agusti C, Soler N: **Bacterial colonisation of distal airways in healthy subjects and chronic lung disease: a bronchoscopic study**. *Eur Respir J* 1997, **10**(5):1137-1144.
